# Quaking but not parkin is the major tumor suppressor in 6q deleted region in glioblastoma

**DOI:** 10.3389/fcell.2022.931387

**Published:** 2022-08-16

**Authors:** Fatma Betul Aksoy Yasar, Takashi Shingu, Daniel B. Zamler, Mohammad Fayyad Zaman, Derek Lin Chien, Qiang Zhang, Jiangong Ren, Jian Hu

**Affiliations:** ^1^ Department of Cancer Biology, The University of Texas M.D. Anderson Cancer Center, Houston, TX, United States; ^2^ The University of Texas MD Anderson Cancer Center UTHealth Graduate School of Biomedical Sciences, Houston, TX, United States; ^3^ Department of Genomic Medicine, The University of Texas MD Anderson Cancer Center, Houston, TX, United States; ^4^ School of Arts and Sciences, University of Rochester, Rochester, NY, United States; ^5^ Department of Investigational Cancer Therapeutics, The University of Texas MD Anderson Cancer Center, Houston, TX, United States

**Keywords:** GBM, parkin, QKI, glioma, glioblastoma

## Abstract

Glioblastoma (GBM) is a high-grade, aggressive brain tumor with dismal median survival time of 15 months. Chromosome 6q (Ch6q) is a hotspot of genomic alterations, which is commonly deleted or hyper-methylated in GBM. Two neighboring genes in this region, *QKI* and *PRKN* have been appointed as tumor suppressors in GBM. While a genetically modified mouse model (GEMM) of GBM has been successfully generated with *Qk* deletion in the central nervous system (CNS), *in vivo* genetic evidence supporting the tumor suppressor function of *Prkn* has not been established. In the present study, we generated a mouse model with *Prkn*-null allele and conditional *Trp53* and *Pten* deletions in the neural stem cells (NSCs) and compared the tumorigenicity of this model to our previous GBM model with *Qk* deletion within the same system. We find that *Qk* but not *Prkn* is the potent tumor suppressor in the frequently altered Ch6q region in GBM.

## Introduction

Gliomas are primary tumors that arise from the supporting glial cells or progenitor cells of the brain and the spinal cord (Cohen and Colman, 2015; Lapointe et al., 2018). The most common and deadliest type of glioma is glioblastoma (GBM), which is a highly aggressive primary brain tumor that has been a therapeutic challenge (Ostrom et al., 2015; Lapointe et al., 2018; Louis et al., 2021). The current standard of care for GBM consists of surgical resection followed by radiotherapy and chemotherapy, upon which the current median survival rate after diagnosis remains at about 14 months (Stupp et al., 2009; Tan et al., 2020; Louis et al., 2021). Molecular mechanisms contributing to tumorigenesis and tumor progression in GBM have long been exploited to identify potential targets for targeted therapies. While various genomic alterations have been associated with GBM, a particular genomic locus that has been deregulated in and associated with GBM is chromosome 6q, particularly 6q25-27 (Ichimura et al., 2006; Cancer Genome Atlas Research Network, 2008; Parsons et al., 2008; Veeriah et al., 2010; Ma et al., 2012; Gao and Smith, 2014). 6q25-27 is a fragile region that is susceptible to instability, evidenced by its highly frequent deletion or methylation in various cancers such as melanoma, colon cancer, gastric cancer, and gliomas (Veeriah et al., 2010; Ma et al., 2012; Gao and Smith, 2014). Moreover, congenital deletion of the 6q27 region leads to a neurological condition named 6q terminal deletion syndrome, which is characterized by mental disability and brain abnormalities (Striano et al., 2006; Backx et al., 2010; Peddibhotla et al., 2015; Bhatta et al., 2020). Besides deleted in over 37% of GBM, chromosome 6q25-27 is also heavily hyper-methylated in ∼20% of GBM, strongly suggesting that potential tumor suppressor(s) resides in this locus (Brennan et al., 2013; Chaligne et al., 2021; Chen et al., 2012; Ichimura et al., 2006; Miyakawa et al., 2000; Mulholland et al., 2006; Veeriah et al., 2010; Yin et al., 2009).

Three neighboring genes residing this locus are *PRKN* (PARKIN), *PACRG* (Parkin Coregulated Gene), and *QKI* (QUAKING), and both PRKN and QKI have been shown to be tumor suppressors in GBM (Gilbert, 2002; Brennan et al., 2013; Darbelli and Richard, 2016; de Castro et al., 2021). QKI is a KH-domain single-stranded nucleic acid-binding protein that modulates various cellular pathways through transcriptional and/or post-transcriptional regulation (Chenard and Richard, 2008; Darbelli and Richard, 2016). We have previously demonstrated that depletion of *Qk* (mouse gene encoding Quaking) along with tumor suppressors *Trp53* and *Pten* in neural precursor cells (NSCs) using Nestin-CreER^T2^ system (QPP) led to GBM formation in mice with a penetrance of over 90%, providing a novel and reliable system to study GBM (Shingu et al., 2017). PARKIN is an E3-ubiquitin ligase that has been named upon the discovery that it is mutated in autosomal recessive juvenile Parkinson Disease (ARJP) (Kitada et al., 1998; Lucking et al., 2000). Located in the 6q25-27 chromosomal region, PRKN is commonly lost/deleted in GBM similar as QKI, and PARKIN protein expression was shown to be downregulated during glioma progression (Cesari et al., 2003; Freije et al., 2004; Veeriah et al., 2010; Xu et al., 2014; Lin et al., 2015; de Castro et al., 2021). However, there is a lack of GBM GEMM models with *Prkn* deletion to provide genetic evidence reinforcing the tumor suppressive role of PARKIN in GBM (Chen et al., 2013). In the current study, we sought to compare the tumor suppressive functions of *Prkn* and *Qk* by deleting them on the same background of *Trp53/Pten* double knockout in NSCs using Nestin-Cre-LoxP system (Tronche et al., 1999).

## Materials and methods

### Mice

Previously we have established Nestin-CreER^T2^ *Pten* ^L/L^ *Trp53* ^L/L^ (PP) mice and Nestin-CreER^T2^ *Qki* ^L/L^ *Pten* ^L/L^ *Trp53* ^L/L^ (QPP) mice (Shingu et al., 2017). Parkin knockout mice were obtained from The Jackson Laboratory (Bar Harbor, ME) (Stock Number: 006582, Strain name: B6.1294-Park2tm1shn/J) (Goldberg et al., 2003). These mice were crossed with PP mice to obtain Nestin-CreER^T2^ *Pten* ^L/L^ *Trp53* ^L/L^ *Prkn* ^−/−^ (PPP) mice. Mice were subcutaneously injected with tamoxifen (200 mg/mouse, postnatal days 7 and 8) to activate Cre-recombinase and induce deletion of *Pten* and *Trp53* in *Nestin*-expressing cells. The mice were housed according to the Association for Assessment and Accreditation of Laboratory Animal Care and NIH standards. The mice were monitored for signs of illness every other day and euthanized and/or harvested when found moribund.

### Brain and tumor harvest and sample preparation

Mice were euthanized with the use of anesthetic or carbon dioxide, followed by cervical dislocation. Brains were removed with or without transcardial perfusion using 4% paraformaldehyde (PFA), followed by post-fixation with formalin at room temperature. Serial sections of 5 μm thickness for paraffin sections were used for subsequent staining applications.

### Antibodies

Antibodies for immunofluorescence (IF) and immunohistochemistry (IHC) were obtained and used as described in the following paragraph. Anti-GFAP (Z0334, rabbit, 1:1,000 for IHC) from DAKO, Agilent Technologies (Carpinteria, CA), anti-CD31 (77699, rabbit, 1:100 for IHC) from CST, Cell Signaling Technology, anti-Ki67 (ab15580, rabbit, 1:200 for IHC) from Abcam, anti-Iba1 (019-19741 rabbit, 1: 200 for IHC and 1:250 for IF) from Wako Chemicals United States, anti-Olig2 (EMD rabbit, 1:200 for IHC) from EMD Millipore. Anti-CD8 (ab209775, rabbit, 1:200 for IF) from Abcam, anti-GrB (AF 1865, goat, 1:100 for IF) from R&D Systems, anti-Tmem119 (ab209064, rabbit, 1:200 for IF) from Abcam, and anti-F4/80 (30325T, rabbit, 1:400 for IF) from Cell Signaling Technology.

### Immunohistochemistry

Formalin-fixed-paraffin-embedded (FFPE) brain tumor sections were deparaffinized at 60°, rehydrated through triple washes with Xylene, 100% EtOH, 95% EtOH, 70% EtOH, 50% EtOH, and ddH2O. After heat-mediated antigen retrieval in 5% citrate-buffer, 3% hydrogen peroxidase was used to quench endogenous peroxidase prior to blocking with 3% bovine serum albumin (BSA) and 1% horse serum (HS). Following blocking, the tumor sections were incubated with primary antibodies overnight at 4° or 2 h at room temperature. The sections were then incubated with horseradish peroxidase (HRP)-conjugated polymer (Biocare Medical, Concord, CA) for 45 min and then with diaminobenzidine using the Ultravision DAB Plus Substrate Detection System (Thermo Fischer Scientific, Waltham, MA) for 5 min at room temperature, followed by hematoxylin staining for 1 min. The tumor sections were then washed, dehydrated, and mounted with coverslips. The light microscopy images were taken with Leica DFC295 Bright Field microscope.

### Immunofluorescence

FFPE brain sections generated from PP, PPP, or QPP animals 4–6 weeks post tamoxifen injection were deparaffinized, rehydrated, and subjected to heat-mediated antigen retrieval in 5% citrate buffer. Slides were then blocked with 1% horse serum (HS) and 3% bovine serum albumin (BSA) and incubated with primary antibodies overnight at 4°. The sections were incubated with secondary antibodies coupled to AlexaFluor dyes (488 or 594, Thermo Fischer Scientific) for 1–2 h at room temperature at a 1:1,000 dilution. Vectashield with DAPI (Vector Laboratories) was used as the mounting medium and cover slips were applied to the stained and mounted sections. The fluorescence images were taken with a Nikon Upright Eclipse Ni-E microscope and cell counting analyses were performed using Fiji/Image J software. Immunofluorescence images were taken from brains harvested from *n* = 3 pairs of mice to be used in quantitative analyses, wherein each data point represents an individual image quantified for antibody-positive cellular signal. Cell numbers per area each represent cell counts in an area of 0.08 mm^2^ within the subventricular zone.

### Statistical analyses

For survival analyses, pairs of Kaplan-Meier survival curves were compared by the log-rank Mantel-Cox test using GraphPad Prism software. For the cell number count statistical analyses of immunofluorescence images, Image J was used to filter the background staining, enhance, and quantify the cellular signal whereas GraphPad Prism software was used to conduct Two-way ANOVA, testing for differences between the three groups/columns. Differences were considered statistically significant when provided *p*-value was less than 0.05.

## Results

### *Qk* deletion but not *Prkn* deletion leads to GBM development on the backdrop of *Pten* and *Trp53* double knockout

**FIGURE 1:**
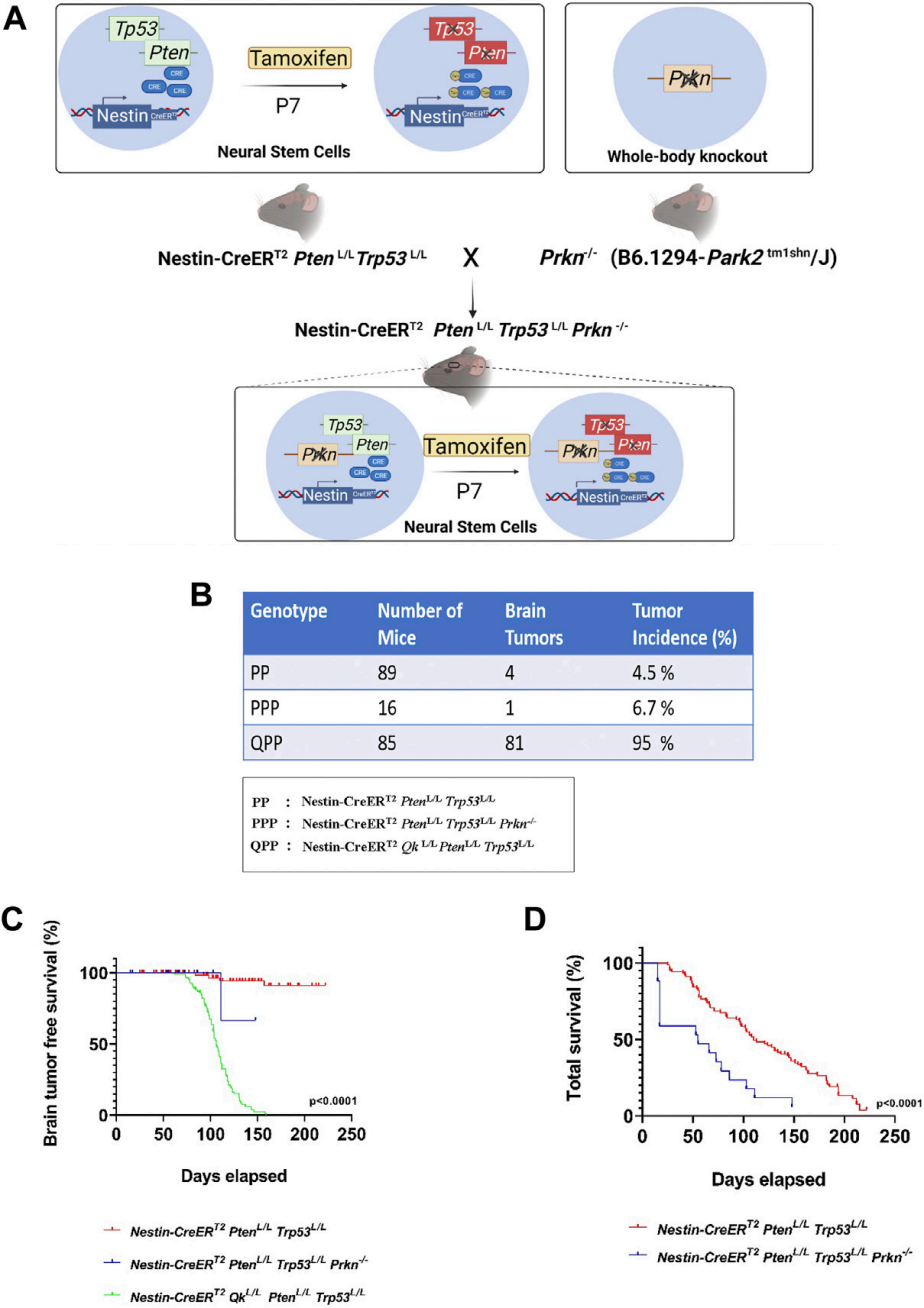
*Prkn* deletion does not lead to GBM development on the backdrop of Pten and Trp53 double knockout. **(A)**. Schematic describing the generation of the PPP genetic model. The illustrations were made using BioRender. **(B)**. The cohort sizes and brain tumor incidences tabulated for PPP, QPP, and PP models. **(C)**. Kaplan-Meier survival curves (long rank test) for PPP, QPP, and PP mice treated with tamoxifen (at P7-P10) demonstrating a significantly (*p* < 0.0001) reduced brain tumor free survival rate for QPP and not for the PP and PPP. **(D)**. Kaplan-Meier survival curves (long rank test) for PPP, and PP mice treated with tamoxifen (at P7-P8) demonstrating a significantly (*p* < 0.0001) reduced total survival rate for the PPP mice compared to the PP mice.

We have previously established Nestin-CreER^T2^ *Qk* ^L/L^ *Pten* ^L/L^ *Trp53* ^L/L^ (QPP) cohort and demonstrated that QPP mice injected with tamoxifen at postnatal day 7 (P7) developed GBM with a penetrance of over 90% and died with a median survival time of ∼105 days, whereas Nestin-CreER^T2^ *Pten* ^L/L^ *Trp53* ^L/L^ (PP) cohort did not develop GBM (Shingu et al., 2017). To test whether *Prkn* deletion could also promote GBM development in the backdrop of *Pten/Trp53* double knockout, we crossed *Prkn*-null allele to Nestin-CreER^T2^ *Pten* ^L/L^ *Trp53* ^L/L^ (PP) mice to generate Nestin-CreER^T2^ *Pten* ^L/L^ *Trp53* ^L/L^ *Prkn* ^−/−^ (PPP) cohort (Figure 1A). Contrary to the QPP mice, neither PP mice nor PPP mice injected tamoxifen at P7 developed GBM, although 4/89 (4.5%) PP mice and 1/15 (6.7%) PPP mice did develop lower grade brain tumors (Figure 1B). In line with this, the glioma-free survival rate of the QPP cohort was significantly lower compared to both PP and PPP cohorts (Figure 1C). Together, these data suggest that, unlike *Qk*, *Prkn* is not a major tumor suppressor in GBM. Of note, total survival rate of the PPP cohort appeared lower than that of the PP cohort, suggesting that Parkin may play an important role in tissue homeostasis (Figure 1D).

### Early premalignant lesions of the QPP mice demonstrated a tumor-permissive microenvironment compared to those of the PP and PPP mice

The tumor microenvironment (TME) has been studied for its critical role in modulating GBM progression, whereas the role of the premalignant brain microenvironment remained elusive (Quail and Joyce, 2017; Huang et al., 2020). Herein, we identified distinct populations of immune cells in the SVZ (subventricular zone) of our PP, PPP, and QPP mice at 6–8 weeks post-injection, before any microscopic tumors could be detected, and explored potential implications of premalignant immune microenvironment profiles on the differential tumorigenic abilities observed in our models.

**FIGURE 2:**
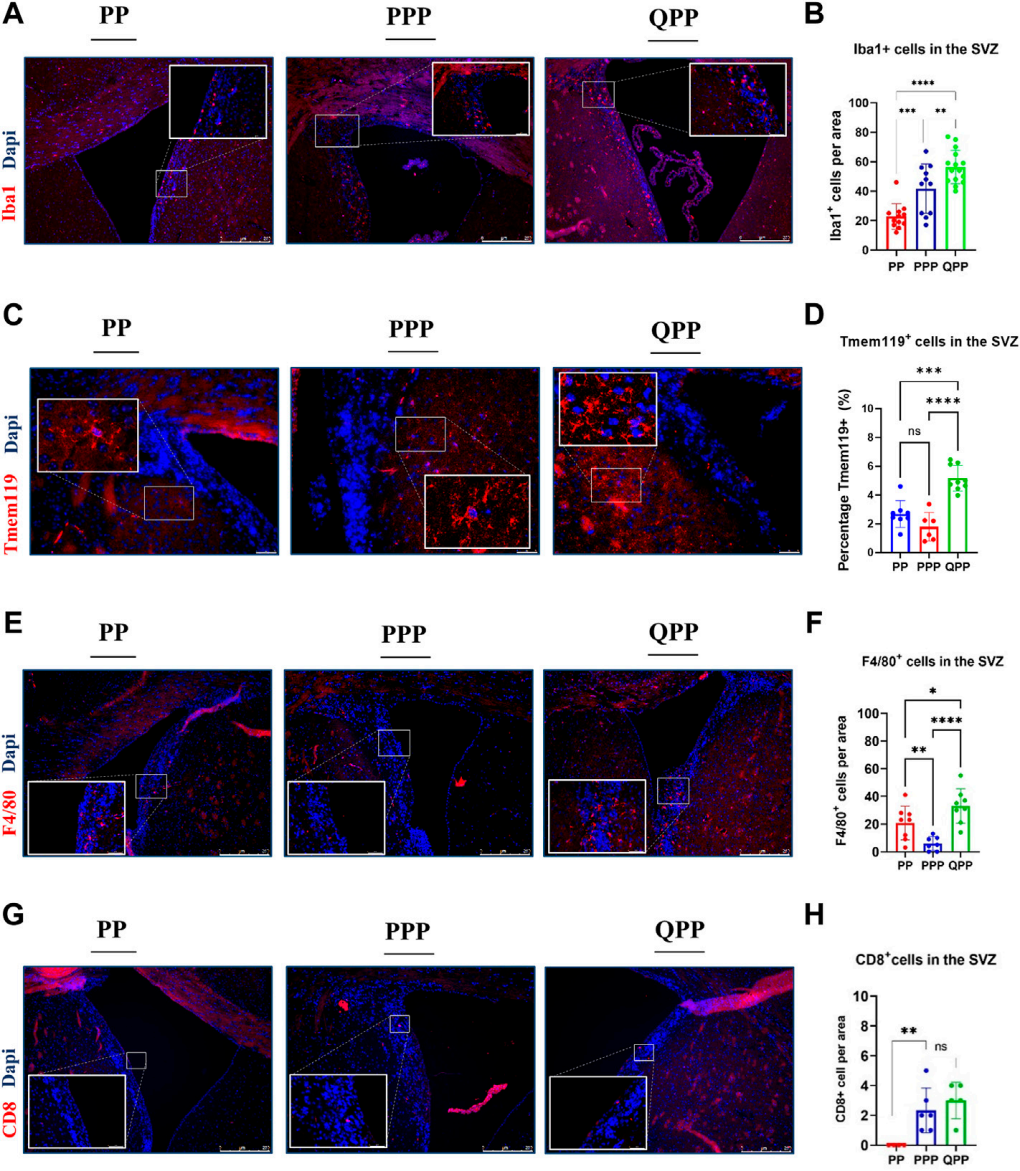
Early premalignant lesions of the QPP mice demonstrated a tumor-permissive microenvironment compared to those of the PP and PPP mice **(A)**. Immunofluorescence staining images of IBA1-positive myeloid cells in the SVZ regions of PP, PPP, and QPP brains, respectively. Scale bars represent 250 μm. **(B)**. Quantification and comparison of IBA1-positive myeloid cell numbers between PP, PPP, and QPP premalignant SVZ regions. **(C)**. Immunofluorescence staining images of TMEM119-positive microglia in the SVZ regions of PP, PPP, and QPP brains, respectively. Scale bars represent 50 μm. **(D)**. Quantification and comparison of TMEM119-positive area percentages between PP, PPP, and QPP premalignant SVZ regions. **(E)**. Immunofluorescence staining images of F4/80-positive macrophages in the SVZ regions of PP, PPP, and QPP brains, respectively. Scale bars represent 250 μm. **(F)**. Quantification and comparison of F4/80-positive macrophage numbers between PP, PPP, and QPP premalignant SVZ regions. **(G)**. Immunofluorescence staining images of CD8-positive lymphocytes in the SVZ regions of PP, PPP, and QPP brains, respectively. Scale bars represent 250 μm. **(H)**. Quantification and comparison of CD8-positive lymphocyte numbers between PP, PPP, and QPP premalignant SVZ regions. (Two-way ANOVA, ns = not significant, * = *p* < 0.05, ** =*p* < 0.01, *** = *p* < 0.001, and **** = *p* < 0.0001).

Tumor-associated macrophages and microglia (TAMs) represent the majority of the immune population within GBM tumors and have been shown to act as immune-suppressors and facilitators of tumor growth (Zhai et al., 2011; Kennedy et al., 2013; Wei et al., 2020). Therefore, we first stained for microglia/macrophage marker Iba1 and found that Iba1^+^ cells were concentrated alongside the SVZ region in all samples (Figure 2A). Iba1^+^ cell numbers were significantly higher in the premalignant SVZ regions of the QPP mice, compared to both PP and PPP brains (Two-way ANOVA, *p* < 0.01) (Figures 2A,B). Moreover, the PPP SVZ regions also appeared to have significantly higher Iba1^+^ cell numbers compared to those of the PP mice (Figures 2A,B). Tissue-resident microglia were also assessed with Tmem119 staining, which demonstrated significantly higher coverage in the QPP premalignant SVZ regions, compared to both PP and PPP (Figures 2C,D).

We also compared murine macrophage marker F4/80^+^ cell numbers in the premalignant SVZ between three models. F4/80^+^ cell numbers were significantly higher in the QPP model compared to PP and PPP, with notably lower rates of infiltration by the peripheral macrophages in the PPP model (Two-way ANOVA, *p* < 0.001) (Figures 2E,F).

In order to assess the infiltration of peripheral lymphocytes, we co-stained pre-malignant SVZ regions of PP, PPP, and QPP with anti-CD8 and anti-Granzyme B antibodies. We detected overall considerably small numbers of CD8^+^ T lymphocytes at this stage in the brains (< 5 cells per 0.08 mm^2^ area). While the CD8-positive cell numbers appeared to be significantly higher in the SVZ of QPP compared to the PP brains, the *Prkn*-deficient PPP pre-malignant SVZ demonstrated comparable numbers (Figures 2G,H). We did not detect any CD8^+^ GrB^+^ double-positive cytotoxic/activated T lymphocytes in any of the pre-malignant samples, in line with the absence of cancerous lesions at this time point. Of note, we also did not detect any CD4^+^ “helper” T lymphocytes or CD4^+^ Foxp3^+^ “regulatory” T-cells in the pre-malignant SVZ regions of our models, accurately representing the low density of these populations in the scRNA-seq analyses of established GBM tumors we recently reported (Zamler et al., 2022).

Together, these findings suggested that the premalignant microenvironment profiles of PP and PPP models appeared to be notably similar to each other when compared to that of the more tumorigenic QPP model. The QPP brain demonstrated an enriched immune suppressive microenvironment prior to tumor formation, characterized by tumor-associated macrophages (TAM), in addition to the potent cell-autonomous tumorigenicity of Qki-deletion detailed in our previous reports.

### Histopathological analyses identified the brain tumor isolated from the PPP cohort as low-grade glioma

As noted above, our PPP cohort has only produced one brain tumor, of which we performed histopathological assessments using H&E staining and immune-histochemistry (IHC). Tumors harvested from our established cohorts PP and QPP were also assessed in comparison, with QPP tumors serving as an established representative for high-grade glioma.

**FIGURE 3:**
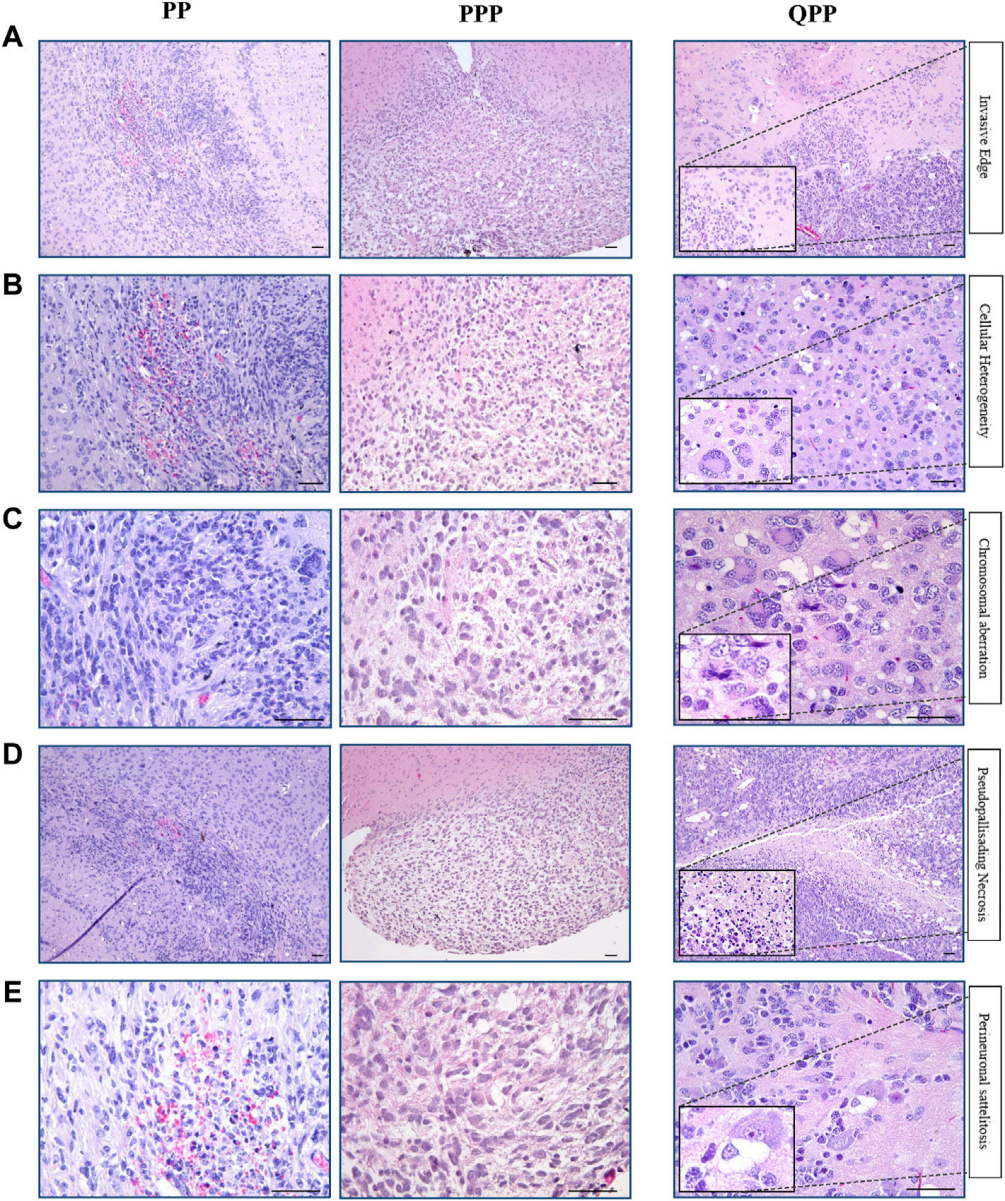
Histopathological analyses identified the brain tumor isolated from the PPP cohort as low-grade glioma. **(A)**. Representative H&E images of tumors harvested from QPP, PPP, and PP cohorts demonstrating invasive edges. **(B)**. Representative H&E images of tumors harvested from QPP, PPP, and PP cohorts indicating intra-tumor cellular heterogeneity. **(C)**. H&E images of tumors harvested from QPP, PPP, and PP cohorts representative of chromosomal aberrations. **(D)**. Representative H&E images of tumors harvested from QPP, PPP, and PP cohorts displaying intra-tumor necrosis. **(E)**. Representative H&E images of tumors harvested from QPP, PPP, and PP cohorts exemplifying perineuronal satellitosis. Scale bars represent 50 μm.

As described in our previous report, QPP tumors exhibit invasive edges, high cellular heterogeneity, frequent chromosomal aberrations, necrosis, and perineuronal satellitosis, all of which suggested that they are high-grade gliomas (grade IV or GBM) (Figures 3A–E) (Shingu et al., 2017). In contrast, the PPP tumor appeared histologically more similar to the low-grade gliomas occasionally isolated from our PP cohort, and lacked the aforementioned characteristics exemplified in the QPP GBM tumors (Figures 3A–D).

**FIGURE 4:**
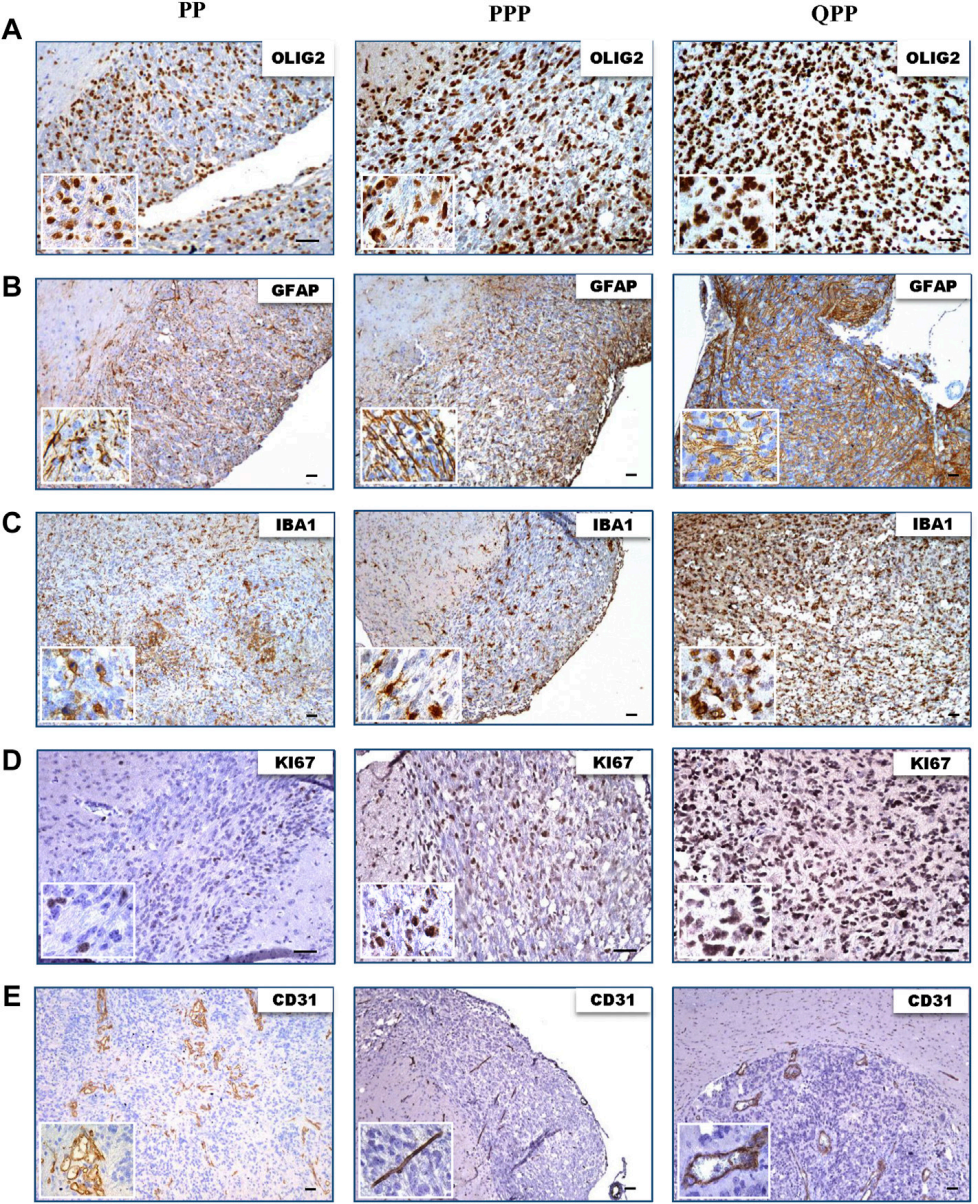
Tumors harvested from all three models express elevated levels of glioma biomarkers. **(A)**. IHC images of tumors harvested from QPP, PPP, and PP cohorts demonstrating OLIG2 expression. **(B)**. IHC images of tumors harvested from QPP, PPP, and PP cohorts demonstrating GFAP expression. **(C)**. IHC images of tumors harvested from QPP, PPP, and PP cohorts demonstrating IBA1 expression. **(D)**. IHC images of tumors harvested from QPP, PPP, and PP cohorts demonstrating KI67 expression. **(E)**. IHC images of tumors harvested from QPP, PPP, and PP cohorts demonstrating CD31 expression. Scale bars represent 50 μm.

We next performed IHC staining to assess the protein expression levels of various glioma markers. All three tumors showed high protein levels for oligodendrocyte lineage marker Olig2, astrocyte lineage marker Gfap, and macrophage/microglia marker Iba1 (Figures 4A–C). All tumors demonstrated proliferation and hyper-vascularity as marked by KI67 and CD31 staining, respectively (Figures 4D,E).

In summary, histopathological analyses of tumor sections obtained from the brains of PP, QPP, and PPP mouse models supported the tumorigenicity and brain-tumor-free survival data. QPP tumors demonstrated a trend of increased staining densities for GBM-indicative protein markers such as Olig2, Gfap, and Iba1, while the PP and PPP tumors displayed histological characteristics similar to the lower-grade gliomas. Nonetheless, a statistical analysis remained out of scope for this study as we could obtain fewer than three brain tumors from the PP and PPP cohorts given their extremely low penetrance.

## Discussion

Chromosome 6q is a fragile region and a genomic alteration hotspot that has been implicated in both neurological diseases and cancer (Miyakawa et al., 2000; Denison et al., 2003; Ichimura et al., 2006; Striano et al., 2006; Weir et al., 2007; Mitsui et al., 2010; Morris et al., 2010; Ma et al., 2012; Bhatta et al., 2020). 95% of the allelic losses in gliomas were found to be affecting chromosome 6q arm, and the alteration rate appeared to be highest in GBM (37%) (Miyakawa et al., 2000). Two prominent genes in Ch6q 25-27 region, *PRKN*, and *QKI*, have both been lost or downregulated in GBM (Cesari et al., 2003; Brennan et al., 2013; Darbelli and Richard, 2016). In this study, we investigated the tumor suppressor role of *Prkn*, on the backdrop of a previously established GEMM system targeting premalignant (PM) NSCs to deplete major tumor suppressors *Trp53* and *Pten* (Shingu et al., 2017). Nestin-CreER^T2^ *Pten* ^L/L^ *Trp53* ^L/*L*^ *Prkn* ^−/−^ (PPP) mice injected at P7 did not form GBM tumors, and the brain tumor-free survival rates appeared similar to the PP animals with only *Trp53* and *Pten* deletions in the same system. Similar to what has been observed in the PP model, the PPP model was also inadequate for high rates of brain tumor formation.

The examination of the immune microenvironment bolstered these findings when we compared the SVZ regions of pre-malignant brains. We found that the QPP model inhabited the highest microglia/macrophage levels, as indicated by Tmem119 and Iba1 staining in the SVZ. This observation was followed by other macrophage markers such as F4/80, which demonstrated a sharp difference between the QPP and PPP SVZ regions, alluding to a scenario that the QPP mice had higher rates of infiltration by the peripheral macrophages compared to the PPP mice, well before the tumorigenesis took place. Lymphocyte infiltration appeared to be noticeably weaker compared to the myeloid lineage, as we have not detected any NK cells (NK1.1^+^) and a very small number of CD8^+^ T cells. These findings demonstrated a clear trend where myeloid immune infiltration into the pre-tumor microenvironment is significantly enriched in QPP mice compared to PPP mice, potentially establishing an environment more susceptible to tumorigenesis.

QKI has long been associated with neurological diseases and cancers, modulating various pathways through both transcriptional and post-transcriptional regulation (Ebersole et al., 1996; Feng and Bankston, 2010; Darbelli and Richard, 2016). Previous TCGA analyses have appointed *QKI* as the common gene shared among the 6q26 chromosome alterations in GBM, alluding to its dominance as the tumor suppressor effector housed in this region (Brennan et al., 2013). Our QPP model demonstrated that loss of Qki leads to the downregulation of the endolysosomal pathway and subsequent receptor recycling, which then enables malignant glioma stem cells to maintain their dedifferentiated state outside their niches for subsequent tumorigenesis (Shingu et al., 2017).

The majority (82%) of chromosome 6q alterations have been found to affect PARKIN expression levels in GBM (Cesari et al., 2003; Veeriah et al., 2010; Xu et al., 2014; de Castro et al., 2021). The tumor suppressor role of Parkin has been implicated with the expression correlation studies where low Parkin expression was associated with poor GBM prognosis (Freije et al., 2004; Wang et al., 2017; de Castro et al., 2021). In the present study, we interrogated the functional role of Parkin as a tumor suppressor and demonstrated a significant difference in tumorigenicity between PPP and QPP models. A double knockout of Qki and Parkin besides Trp53 and Pten deletion using the same system warrants further exploration to inquire about a potential compound effect in GBM pathology. Interestingly, despite a low penetrance for brain tumor formation, the total survival rate of the PPP cohort nonetheless appeared to be lower than the PP cohort. This unprecedented premature lethality phenotype of our Parkin-null animals could be explained by the breeder mouse strain/background differences or Nestin-CreER^T2^ expression and consequent loss of Pten and p53 outside of the brain that could have exacerbated the original Parkin knockout phenotype (Noda et al., 2020).

While *PRKN* gene has been reported to be frequently mutated/lost in GBM, one possible scenario is that deletions on the *PRKN* gene exert indirect effects on QKI, owing to disruption of regulatory regions and long-range chromatin interactions that modulate QKI expression levels. One such example has already been well established in *qk* ^*v*^ (quaking viable) mice, where a >1 Mb deletion on chromosome 17 encompasses *Prkn* coding sequence as well as ∼1 kb upstream of the *Qk* gene (Ebersole et al., 1996; Lockhart et al., 2004; Sidman et al., 1964). The deletion of a putative tissue-specific enhancer in this region leads to a significant reduction of Qki expression in oligodendrocytes, leading to severe hypomyelination in the CNS. The phenotype in mutant mice was later confirmed to be solely caused by Qki loss and was not recapitulated by *Prkn*-null animals (Wolf and Billings-Gagliardi, 1984; Ebersole et al., 1996; Mata et al., 2004; Perez and Palmiter, 2005; Darbelli et al., 2016; Shingu et al., 2017). Similarly, somatic deletions within the *PRKN* gene sequence could potentially disrupt the regulatory sequences and tissue-specific enhancers acting on *QKI* gene expression, leading to an underestimation of *QKI* alterations in GBM while overestimating the tumor suppressor function of *PRKN*. Mapping of long-range chromatin interactions and identification of putative regulatory regions within Ch6q using functional and genetic assays will provide critical insights on this matter.

## Data Availability

The original contributions presented in the study are included in the article, further inquiries can be directed to the corresponding author.
